# Treating dysfunctional one-carbon metabolism in glaucoma

**DOI:** 10.4103/NRR.NRR-D-25-01089

**Published:** 2025-12-30

**Authors:** James R. Tribble, Pete A. Williams

**Affiliations:** Department of Clinical Neuroscience, Division of Eye and Vision, St. Erik Eye Hospital, Karolinska Institutet, Stockholm, Sweden

The majority of our daily activities and routines are highly dependent on vision. What we experience as our vision arises from the detection and encoding of visual signals in the retina, which are then interpreted in the brain. This interpretation has the benefit of providing a level of constancy to what we experience as vision but also limits our ability to perceive subtle decline in our own vision. This underlies a challenge in the early diagnosis and treatment of sight threatening diseases such as glaucoma. Glaucoma is characterized by the progressive dysfunction and death of retinal ganglion cells (RGCs), the neurons that relay visual information from the retina to the brain via their axons, which bundle to form the optic nerve. To be diagnosed with glaucoma requires a degree of RGC loss sufficient to produce perceptual changes to vision. In the majority of cases, the continued progressive loss of vision is gradual but there are subtypes, which more rapidly reach blindness. Consequently, up to 40% of treated patients will reach blindness in at least one eye in their lifetime (Peters et al., 2014). Given that current estimates place the number of people with glaucoma at over 100 million globally (Tham et al., 2014), glaucoma is a major health concern. The main risk factors for glaucoma are age, genetics, and high intraocular pressure (IOP). IOP lowering is the only proven glaucoma treatment; however, a large percentage of patients do not respond to IOP lowering (i.e., IOP remains high despite treatment), continue to lose vision despite IOP control (i.e., reducing IOP does not prevent progression), or are normotensive (develop glaucoma despite having IOPs in the normal range). There is, therefore, a large clinical need for neuroprotective therapies for glaucoma that act independently of IOP (Tribble et al., 2023).

**Figure 1:**
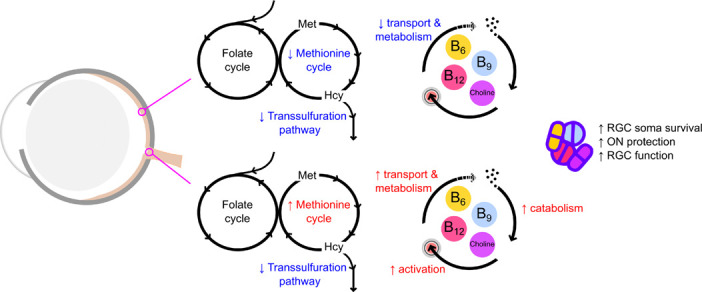
Treating dysfunctional one-carbon metabolism in glaucoma. One-carbon metabolism is disrupted in the retina and ONH in glaucoma. In the retina, the downregulation of genes in the methionine cycle and transsulfuration pathway is consistent with an increase in retinal homocysteine. This may be linked to a downregulation of genes associated with the transport and metabolism of one-carbon metabolism cofactors and precursors. In comparison, the ONH demonstrates an upregulation in the methionine cycle and an increase in the transport, metabolism, activation, and catabolism of these one-carbon metabolism cofactors and precursors, which may reflect the pro-inflammatory environment of the ONH. Supplementing these one-carbon metabolism cofactors and precursors provided neuroprotection to RGC somas, axons, and function (Tribble et al., 2025). Created with Inkscape (https://inkscape.org/). Hcy: Homocysteine; Met: methionine; ON: optic nerve; ONH: optic nerve head; RGC: retinal ganglion cell.

In the search for early features of glaucoma, metabolic function is increasingly being recognized as a key determinant of RGC degeneration and survival (Casson et al., 2021; Tribble et al., 2023). We have previously identified metabolic dysfunction in glaucoma and developed metabolomic approaches to quantify changes to small molecular weight metabolites (Harder et al., 2020; Tribble et al., 2021). These studies identified that metabolic dysfunction occurs prior to detectable neurodegeneration of RGCs. We identified a significant elevation of homocysteine in ocular hypertension (OHT) in rats (Tribble et al., 2021). Homocysteine is a non-coding amino acid with a central role in one-carbon metabolism (an important anabolic hub) with established roles in various diseases, including cardiovascular disease, diabetes, and vascular dementias, through direct toxic effects from its elevation. Whilst elevated homocysteine had been correlated to glaucoma across various small-scale studies (typically ~30 patients per arm for blood homocysteine, and ~150 patients per arm for gene variants associated with elevated homocysteine) (Xu et al., 2012), whether homocysteine has a significant role in glaucoma was unestablished. We hypothesized that homocysteine elevation in the retina could be an important contributing feature to RGC degeneration and may be linked to dysfunctional one-carbon metabolism (Tribble et al., 2025). Using a rat model of ocular hypertensive glaucoma, where we could artificially elevate homocysteine in addition to OHT, we demonstrated that there was no synergistic effect from these two insults, supporting that elevated homocysteine did not predispose to greater RGC loss in glaucoma. Using large-scale population data from the UK biobank linking known genetic determinants of elevated homocysteine with ophthalmic imaging (30,000–200,000 individuals), and a secondary analysis of the UKGTS clinical trial (74 strictly phenotyped patients per arm) where we could compare visual field outcome to serum homocysteine levels, we demonstrated that elevated homocysteine does not increase the risk of glaucoma nor significantly worsen glaucoma related outcomes. Systemically high homocysteine had little impact in driving disease, and the local elevation we detected in the rat retina was likely linked instead to dysfunctional one-carbon metabolism (Tribble et al., 2025). By quantifying the central enzymes related to homocysteine in this pathway, we demonstrated at the gene and protein levels that OHT rats had changes consistent with homocysteine elevation in the retina and optic nerve (**Figure 1**). Using gene sequencing data spanning from early to severe disease course in the DBA/2J mouse (a mouse model of an inherited ocular hypertensive glaucoma), we demonstrated that differential expression of genes relative to controls was far more widespread across one-carbon metabolism, affecting the methylation cycle, folate cycle, transsulfuration pathway, and numerous immediately adjacent pathways in the retina and optic nerve head (**Figure 1**). Upregulation in the methylation cycle in the optic nerve head was contrasted with its downregulation in the retina (Tribble et al., 2025), potentially reflecting the concentrated pro-inflammatory responses at the optic nerve head and explaining findings of increased DNA methylation under OHT in rats (Husain et al., 2024). Changes to these pathways were not restricted to one cell type, but were present in RGCs, microglia, and infiltrated monocytes. This suggests that anabolic disturbance is potentially an early and sustained feature of glaucoma. Supporting this, induced pluripotent stem cells from glaucoma patients, when differentiated into RGCs, also demonstrated differential expression of some one-carbon metabolism genes, supporting the potential for intrinsic vulnerabilities to disturbance of these pathways in glaucoma patients (Tribble et al., 2025). These may be further exacerbated with additional disease stressors, such as OHT, assuming the same dysregulation we see in animal models occurs in humans.

One-carbon metabolism is reliant on methyl cobalamin (a form of vitamin B_12_) and pyridoxal 5′-phosphate (an active form of vitamin B_6_) as co-factors in the methylation cycle, folate cycle, and/or transsulfuration pathway. Vitamin B_9_ (folic acid) is the precursor fed into the folate cycle, which is coupled to the methyl cycle by provision of a methyl donor for homocysteine to L-methionine conversion. Choline serves as a precursor for betaine, which can function as an alternative methyl provider (**Figure 1**). We identified that many of the one-carbon metabolism enzymes that were dysregulated were those that directly interacted with these co-factors or were involved in the conversion of these precursors. Further examination revealed wider gene dysregulation of the metabolism, catabolism, transport, and activation of these co-factors and precursors (**Figure 1**), which were again present in RGCs, microglia, monocytes, and human RGCs from induced pluripotent stem cells. These data supported the hypothesis that the retina and optic nerve experience a deficit in vitamin metabolism as part of glaucoma pathology, which may be causally related to, or compound, one-carbon metabolism dysfunction (Tribble et al., 2025). By supplementing higher doses of these vitamins (vitamins B_6_, B_9_, B_12_, and choline) than are contained in the diet, we demonstrated that this was sufficient to provide significant neuroprotection against RGC loss in glaucoma. Rats with acute OHT (average IOP of ~35 mmHg, average RGC loss of 40% over 2 weeks) had moderate protection against RGC loss (down to 24% on average) with improved optic nerve profiles (reduced axon loss and stress responses, but not inflammation) independent of IOP. This was repeated in a more chronic mouse model (average IOP of ~18 mmHg, average RGC loss of 17% over 7 weeks) with complete neuroprotection, as well as a protection of RGC function (as measured by the RGC specific positive scotopic threshold response waveform of the electroretinogram) (**Figure 1**). To determine if there may be hints of a protective effect of higher intake of these vitamins in the population, we returned to the UK biobank and analyzed dietary recall data in relation to glaucoma risk and measures of inner retinal thickness from ophthalmic imaging, but no clear associations were identified. We cannot determine whether this reflects a lack of association in humans or a weakness of the dietary recall data as an appropriate surrogate of long-term intake of these vitamins. Nonetheless, the robust protection achieved in the animal models supports the potential of these one-carbon metabolism related vitamins as a translatable therapy to provide neuroprotection in glaucoma (Tribble et al., 2025).

Since these vitamins are poised for rapid clinical translation, providing safety and efficacy is assessed before advising patients, we initiated a clinical trial to test the safe, human-adjusted doses in existing glaucoma patients (NCT06885827; Golpour et al., 2025). This is a randomized, open label Phase II trial, which will compare vitamins B_6_, B_9_, B_12_, and choline in combination with clinician’s best choice primary treatment (IOP lowering) versus clinician’s best choice primary treatment (IOP lowering) only. The trial will determine if this cocktail can improve or protect against changes in RGC function over 12-months (photopic negative response of the electroretinogram, the primary endpoint). Secondary endpoints will assess other visual function measures and retinal structure via OCT imaging. By collecting blood samples for metabolomic analysis, we also aim to identify metabolic changes that occur as a result of treatment and identify whether these treatments exert any systemic metabolic benefits.

These findings of one-carbon metabolism dysfunction and the neuroprotective effects of one-carbon metabolism related cofactors and precursor vitamins B_6_, B_9_, B_12_, and choline represent an exciting new development in the pursuit of new treatments for glaucoma. While these can be taken rapidly to trial thanks to good existing safety data, the continued exploration of the role of one-carbon metabolism in neuronal health and neurodegeneration will be necessary to improve these treatments long-term. It remains to be determined what the downstream consequences of one-carbon metabolism dysfunction are, and mechanistic studies probing these pathways will be required. Similarly, we lack direct evidence of vitamins B_6_, B_9_, B_12_, and choline working through a one-carbon metabolism-dependent mechanism, and further experiments testing this hypothesis will be necessary to refine this treatment.

*This work was supported by Vetenskapsrådet 2022-00799 and the Ulla and Ingemar Dahlberg Foundation (to PAW).*
